# Apolipoprotein E gene polymorphism modifies fasting total cholesterol concentrations in response to replacement of dietary saturated with monounsaturated fatty acids in adults at moderate cardiovascular disease risk

**DOI:** 10.1186/s12944-017-0606-3

**Published:** 2017-11-23

**Authors:** Israa M. Shatwan, Michelle Weech, Kim G. Jackson, Julie A. Lovegrove, Karani S. Vimaleswaran

**Affiliations:** 1 Hugh Sinclair Unit of Human Nutrition and Institute for Cardiovascular and Metabolic Research (ICMR), Department of Food & Nutritional Sciences, University of Reading, Whiteknights, PO Box 226, Reading, RG6 6AP UK; 2 Food and Nutrition Department, Faculty of Home Economics, King Abdulaziz University, Jeddah, Saudi Arabia

**Keywords:** Apolipoprotein E polymorphism, Saturated fatty acids, Monounsaturated fatty acids, Total cholesterol, Gene-diet interaction, DIVAS

## Abstract

**Background:**

Consumption of ≤10% total energy from fat as saturated fatty acids (SFA) is recommended for cardiovascular disease risk reduction in the UK; however there is no clear guidance on the optimum replacement nutrient. Lipid-associated single-nucleotide polymorphisms (SNPs) have been shown to modify the lipid responses to dietary fat interventions. Hence, we performed a retrospective analysis in 120 participants from the Dietary Intervention and VAScular function (DIVAS) study to investigate whether lipoprotein lipase (*LPL*) and apolipoprotein E (*APOE*) SNPs modify the fasting lipid response to replacement of SFA with monounsaturated (MUFA) or n-6 polyunsaturated (PUFA) fatty acids.

**Methods:**

The DIVAS study was a randomized, single-blinded, parallel dietary intervention study performed in adults with a moderate cardiovascular risk who received one of three isoenergetic diets rich in SFA, MUFA or n-6 PUFA for 16 weeks.

**Results:**

After the 16-week intervention, a significant diet-gene interaction was observed for changes in fasting total cholesterol (*P* = 0.001). For the *APOE* SNP rs1064725, only TT homozygotes showed a significant reduction in total cholesterol after the MUFA diet (*n* = 33; −0.71 ± 1.88 mmol/l) compared to the SFA (*n* = 38; 0.34 ± 0.55 mmol/l) or n-6 PUFA diets (*n* = 37; −0.08 ± 0.73 mmol/l) (*P* = 0.004). None of the interactions were statistically significant for the other SNPs.

**Conclusions:**

In summary, our findings have demonstrated a greater sensitivity of the *APOE* SNP rs1064725 to dietary fat composition, with a total cholesterol lowering effect observed following substitution of SFA with MUFA but not n-6 PUFA. Further large intervention studies incorporating prospective genotyping are required to confirm or refute our findings.

**Trial registration:**

The trial was registered at www.clinicaltrials.gov as NCT01478958.

**Electronic supplementary material:**

The online version of this article (10.1186/s12944-017-0606-3) contains supplementary material, which is available to authorized users.

## Background

A high consumption of saturated fatty acids (SFA) has been linked to increased circulating concentrations of low-density lipoprotein cholesterol (LDL-C) [1], and is consequently associated with an increased cardiovascular disease (CVD) risk [2]. Therefore, dietary guidelines have focused on reducing intakes of SFA by ≤10% of total energy (TE) for CVD risk reduction [3]. It is important to consider the nutrients that replace SFAs and previous findings have suggested substitution of SFA with unsaturated fatty acids may provide a greater reduction in CVD risk than refined carbohydrates [4, 5]. In particular, replacement with cis-monounsaturated fatty acids (MUFA) or polyunsaturated fatty acids (PUFA) has been shown to significantly lower fasting total and LDL-C [6, 7]. However, the inter-individual variability in fasting plasma lipid responses to dietary fat intake is high; evidence supports that this is influenced by lipid-associated single-nucleotide polymorphisms (SNPs) such as apolipoprotein E (*APOE*) and lipoprotein lipase (*LPL*) genotypes [8–10].

Several genes are involved in the regulation of lipid transport and metabolism [11]. Among these, the most commonly studied genes with central roles in lipid metabolism are *LPL* and *APOE* [12–14]. The *LPL* SNPs, rs320 (*HindIII*) and rs328 (*S447X*), have been proposed as important genetic determinants of the inter-individual variability in fasting and postprandial triacylglycerol (TAG) concentrations and high-density lipoprotein cholesterol (HDL-C) [15–17]. Increased activity of the LPL enzyme in minor allele carriers of *LPL* SNP rs328 has been shown to be associated with lower plasma TAG and higher HDL-C levels [18]. To date, there has only been one study reporting an interaction between *LPL* rs328 and n-6 PUFA intake on fasting TAG concentrations [19]. The effect of genetic variations of *APOE* on lipid concentrations (i.e. LDL-C) [20–23] and the effect of the *APOE* polymorphisms on the circulating lipid response to dietary fat (i.e. SFA and MUFA) have been previously demonstrated; however, the findings have been inconsistent [24–26]. In addition, investigations into other SNPs of the *APOE* gene are limited.

In the Dietary Intervention and VAScular function (DIVAS) study, the isoenergetic replacement of 9.5–9.6% TE from SFA with *cis* MUFA or n–6 PUFA for 16 weeks in 195 adults at moderate CVD risk resulted in significant reductions of 8.4% and 9.2%, respectively, in total cholesterol, and 11.3% and 13.6%, in LDL-C, in the fasted state [6]. To investigate whether genetic polymorphisms contributed to the observed reductions in total and LDL-C, a retrospective post hoc analysis of the DIVAS study was performed. We examined whether the two *LPL* and seven tagging SNPs (TagSNPs) in the *APOE* gene modified the response of the fasting lipid profile to substitution of SFA with MUFA or n-6 PUFA in this study population at moderate CVD risk.

## Participants and methods

### Study participants

A detailed description of the DIVAS study design and methods has been reported elsewhere [6, 27]. Briefly, participants were recruited from Reading, UK and the surrounding area in three cohorts between November 2009 and July 2012. Participants were aged between 21 and 60 years and were all non-smoking men and women with a moderate risk of CVD. A scoring tool [27] was used to determine CVD risk based on the presence of single or multiple risk factors, including elevated fasting total cholesterol or fasting glucose, raised blood pressure, low HDL-C, being overweight or obese, and/or having a family history of premature myocardial infarction or type 2 diabetes. Eligible participants had a risk score of ≥ 2 combined points, reflecting a moderate CVD risk (≥50% above the population mean). Other criteria for exclusion were the presence of abnormal fasting blood biochemistry, taking dietary supplements or the use of medications that affect lipid metabolism or hypertension, and having inflammatory disorders. The West Berkshire Local Research ethics committee (09/ H0505/56) and the University of Reading Research Ethics Committee (09/40) gave a favourable ethical opinion for conduct. The trial was registered at www.clinicaltrials.gov as NCT01478958. All participants provided written informed consent before participating. In our retrospective analysis, 120 of the 195 participants who completed the DIVAS study consented to genetic analysis, and were included in the present study.

### Study design and diets

The DIVAS study was a randomized, single-blinded, parallel design. The participants completed 16 weeks of dietary intervention, receiving one of three isoenergetic diets based on a minimization program that matched for age, sex, body mass index (BMI), and total CVD risk score. The three intervention diets (%TE derived from SFA:MUFA:n-6 PUFA) were either rich in SFAs (17:11:4), MUFAs (9:19:4), or n-6 PUFAs (9:13:10). Given that dietary guidelines recommend limiting n-6 PUFA intake to ≤10% TE [28], SFA were replaced with 6% TE n-6 PUFA and 2% TE MUFA in the n-6-PUFA-rich diet. The total fat content of all three intervention diets was 36% TE, and intakes of protein, carbohydrates, and n-3 PUFA were unchanged. A greater SFA exchange than the target 8% TE was achieved: SFA vs MUFA was 9.5% TE and SFA vs n-6 PUFA was 9.6% TE [27].

Further details of the dietary intervention procedure and measures of compliance have been published previously [27]. In summary, these interventions were based on a flexible food-exchange model to achieve the target fatty acid intakes in free-living individuals for 16 weeks. Participants, who were randomly assigned to one of three intervention diets, replaced routinely consumed sources of exchangeable fats with study foods. The study foods included spreads, oils, dairy products, and commercially available snacks of a specific fatty acid composition. Specially formulated spreads (80% total fat) and oils (Unilever Research and Development) were used for the MUFA-rich diet (refined olive oil and olive oil/rapeseed oil blended spread) and n–6 PUFA-rich diet (safflower oil and spread). Butter (Wyke Farm) was used as both a spread and oil replacement in the SFA-rich diet.

### Anthropometric measurements and biochemical parameters

Clinical visits took place at the Hugh Sinclair Unit of Human Nutrition, University of Reading, during weeks 0 (baseline; V1) and 16 (after intervention; V2) as described elsewhere [6]. Alcohol and aerobic exercise were avoided 24 h before visits. Participants consumed a provided low-fat meal the evening before visits and fasted for 12 h, only drinking low-nitrate water during this time. Height and weight was recorded at the study visits at weeks 0 and 16 in order to calculate BMI. Height was recorded to the nearest 0.5 cm using a wall-mounted stadiometer and weight was measured using a digital scale (Tanita Europe) using standard settings (normal body type and 1 kg for clothing).

At weeks 0 and 16, fasting blood samples collected into a serum separator vacutainer and a K3EDTA-containing vacutainer (week 0 only) were used for the measurement of the fasting lipid profile and isolation of the buffy coat, respectively. The K3EDTA-containing vacutainer was kept on ice for 30 min before the blood tubes were centrifuged at 1700 *g* for 15 min at 20 °C (for serum) and 4 °C (for plasma). The buffy coat was stored at −20 °C and serum samples stored at −80 °C prior to analysis of total cholesterol, TAG, and HDL-C, and glucose (baseline only) concentrations using an autoanalyzer (reagents and analyzer: Werfen UK Ltd). Fasting LDL-C was estimated using the Friedewald formula [29]. With the use of A/A grade automated oscillometric ambulatory blood pressure (ABP) monitors (A&D Instruments Ltd.), baseline 24 h ABP was measured every 30 min from 07:00 to 21:59 and every 60 min from 22:00 to 06:59, approximately 48 h before the clinical visits.

### SNP selection and genetic analysis

The *APOE* gene is located on chromosome 19q13.32 and comprises of four exons, which are transcribed into the 1180 nucleotides long *APOE* mRNA. The seven tagSNPs for the *APOE* gene were chosen based on International HapMap Phase II collected in individuals of Northern and Western European ancestry (CEU) (HapMap Data release 27 Phase 2 + 3, Feb 09, NCBI B36 assembly, dbSNP b126). The Haploview software V3.3 (https://www.broadinstitute.org/haploview/haploview) was used to assess the linkage disequilibrium structure between SNPs. Tagger software was used to select tag SNPs with the ‘pairwise tagging only’ option. Two criteria were used to filter the SNPs included in the analysis - minor allele frequency ≥ 5% and Hardy–Weinberg equilibrium *P*-value >0.01. Seven tagSNPs (rs405509 (G > T) [30, 31], rs1160985 (C > T) [32], rs769450 (G > A) [33], rs439401 (C > T) [34], rs445925 (G > A) [35], rs405697 (G > A) [36], and rs1064725 (T > G)) representing the entire common genetic variations across the *APOE* gene were selected for the study. In addition, the two commonly studied *LPL* SNPs, rs320 and rs328, were chosen. In total, nine common SNPs were examined in the present study.

DNA was extracted from the buffy coat using a QIAamp DNA blood kit (QIAGEN) and stored at −20 °C. The genotyping of the *LPL* and *APOE* SNPs was outsourced to LGC Genomics (http://www.lgcgroup.com/services/genotyping), which employs the competitive allele-specific PCR-KASP® assay.

### Statistical analysis

The data are presented as mean ± standard deviation (SD) in the tables and text, and as standard error in the figure. The normal distribution was tested for variables, and none of the variables skewed the distribution. The minor allele frequency was calculated by counting. The dominant models were a better fit for SNPs rs320, rs328, rs769450, rs439401, rs445925, rs405697, and rs1064725; thus, homozygosity for the common allele was compared with carriers of the minor allele (heterozygous and homozygous for the minor allele) in the analysis. The additive model was applied for SNPs rs405509 and rs1160985 (major allele homozygotes vs. heterozygotes vs. minor allele homozygotes). The genotype distributions of the nine SNPs at the *LPL* and *APOE* genes were in Hardy-Weinberg equilibrium (*P* > 0.05). Independent t-tests were used to compare means between men and women at baseline. The baseline and over 16 weeks’ associations of the selected SNPs with continuous phenotypes were evaluated by the general linear model (GLM). Moreover, potential interactions between genotype and dietary intervention on 16-week changes of lipids were analyzed by using GLM, where an interaction term was included in the model. Potential confounders associated with the outcomes were adjusted in all GLM analyses (i.e. age, sex, BMI, and ethnicity). When a significant diet x genotype interaction was found, data were split by genotype group and analyzed further by using GLM. A Bonferroni correction was applied and the significant *P* value was 0.0013 (0.05/9 SNPs*4 lipid outcomes). For all analyses, the statistical package SPSS version 22.0 (SPSS, Chicago, IL, USA) was used.

**Table 1:** Baseline characteristics of study participants in the whole group and stratified by sex

Characteristics	Whole group (*N* = 120)	Men (*N* = 54)	Women (*N* = 66)	*P* value
Age	47 ± 9	48 ± 9	46 ± 9	0.37
BMI	26.3 ± 3.9	26.7 ± 3.6	26.2 ± 4.3	0.58
Systolic blood pressure	122 ± 10	126 ± 9	120 ± 9	0.01
Diastolic blood pressure	75 ± 7	77 ± 7	74 ± 7	0.03
Total cholesterol	5.58 ± 1.11	5.75 ± 1.15	5.45 ± 1.06	0.16
TAG	1.28 ± 0.60	1.62 ± 0.61	1.02 ± 0.44	<0.0001
HDL-C	1.54 ± 0.35	1.36 ± 0.32	1.68 ± 0.32	<0.0001
LDL-C	3.79 ± 0.99	4.06 ± 1.01	3.57 ± 0.93	0.01
Glucose	5.11 ± 0.42	5.23 ± 0.44	5.03 ± 0.39	0.02

Data shown are represented as means ± SD, wherever appropriate. *P* values for the differences in the means between men and women. *P* values were calculated by using independent t-test

*BMI* body mass index, *TAG* triacylglycerol, *HDL-C* high- density lipoprotein cholesterol, *LDL-C* low- density lipoprotein cholesterol

## Results

In this retrospective analysis, 120 participants (mean age, 47 ± 9 years; BMI, 26.4 ± 4.0 kg/m^2^) were included. Table 1 illustrates the main characteristics of the study participants stratified according to sex at baseline. Women had significantly lower levels of fasting TAG (*P* < 0.0001), LDL-C (*P* = 0.01), glucose (*P* = 0.02), blood pressures (*P* ≤ 0.03), and higher levels of fasting HDL-C (*P* < 0.0001) compared to men.

The genotype distributions of both *LPL* and *APOE* polymorphisms are shown in Table 2. The participants’ characteristics at the beginning of the dietary interventions (week 0) are presented in Table 3 according to *LPL* and *APOE* genotypes. None of the variables (including fasting TAG, total cholesterol, LDL-C, and HDL-C) were associated with the *LPL* and *APOE* SNPs at baseline. After 16 weeks of intervention, there was also no significant association of the *LPL* and *APOE* SNPs with changes in the lipid outcomes after Bonferroni correction (Tables 4, 5 and Additional file 1: Tables S1-S3).

**Table 2:** Genotype and minor allele frequencies of the SNPs at *LPL* and *APOE* genes in the DIVAS cohort of adults with moderate CVD risk

SNP	MAF	Common homozygous N (%)	Heterozygous N (%)	Rare homozygous N (%)
*LPL*				
rs320 (T > G)	0.30	56 (0.47)	54 (0.45)	9 (0.07)
rs328 (C > G)	0.13	89 (0.74)	30 (0.25)	1 (0.008)
*APOE*				
rs405509 (G > T)	0.47	31 (0.25)	64 (0.53)	25 (0.20)
rs769450 (G > A)	0.37	44 (0.36)	61 (0.50)	15 (0.12)
rs439401 (C > T)	0.35	50 (0.42)	52 (0.44)	16 (0.13)
rs445925 (G > A)	0.11	91 (0.77)	25 (0.21)	1 (0.008)
rs405697 (G > A)	0.25	70 (0.58)	38 (0.31)	11 (0.09)
rs1160985 (C > T)	0.45	33 (0.27)	64 (0.53)	23 (0.19)
rs1064725 (T > G)	0.05	108 (0.90)	12 (0.10)	–

*MAF* minor allele frequency, *LPL* lipoprotein lipase, *APOE* apolipoprotein E

**Table 3:** Baseline characteristics of the DIVAS study participants according to the *LPL* and *APOE* genotypes

SNP	Age	Sex (M/F)	TAG (mmol/l)	HDL-C (mmol/l)	LDL-C (mmol/l)	Total cholesterol (mmol/l)
*LPL*						
rs320						
TT	46 ± 9	27/29	1.28 ± 0.63	1.54 ± 0.41	3.73 ± 0.96	5.52 ± 1.15
T/G	48 ± 9	27/36	1.27 ± 0.57	1.53 ± 0.31	3.81 ± 1.01	5.59 ± 1.07
P value			0.83	0.41	0.71	0.53
rs328						
CC	46 ± 9	42/47	1.29 ± 0.63	1.52 ± 0.37	3.72 ± 1.01	5.49 ± 1.17
C/G	48 ± 10	12/19	1.23 ± 0.47	1.58 ± 0.27	3.96 ± 0.88	5.79 ± 0.83
*P* value			0.51	0.93	0.42	0.49
*APOE*						
rs405509						
GG	47 ± 10	17/14	1.41 ± 0.63	1.42 ± 0.39	3.71 ± 1.01	5.41 ± 1.20
GT	47 ± 9	21/43	1.18 ± 0.57	1.58 ± 0.31	3.77 ± 1.00	5.58 ± 1.05
TT	45 ± 11	16/9	1.34 ± 0.61	1.55 ± 0.38	3.85 ± 0.94	5.67 ± 1.12
*P* value			0.57	0.24	0.81	0.70
rs769450						
GG	47 ± 10	24/20	1.30 ± 0.61	1.57 ± 0.37	3.88 ± 0.96	5.71 ± 1.11
G/A	46 ± 9	30/46	1.25 ± 0.59	1.51 ± 0.34	3.71 ± 1.00	5.47 ± 1.10
*P* value			0.89	0.24	0.99	0.74
rs439401						
CC	48 ± 9	25/25	1.37 ± 0.61	1.53 ± 0.36	3.88 ± 0.84	5.68 ± 0.95
T allele	46 ± 10	29/39	1.22 ± 0.59	1.53 ± 0.34	3.69 ± 1.07	5.47 ± 1.19
*P* value			0.43	0.95	0.54	0.54
rs445925						
GG	46 ± 10	40/51	1.26 ± 0.58	1.53 ± 0.35	3.72 ± 1.00	5.50 ± 1.13
A allele	49 ± 8	13/13	1.36 ± 0.67	1.56 ± 0.38	3.97 ± 1.00	5.80 ± 1.04
*P* value			0.95	0.77	0.64	0.61
rs405697						
GG	47 ± 9	35/35	1.32 ± 0.62	1.52 ± 0.39	3.77 ± 0.91	5.56 ± 1.01
A allele	46 ± 10	19/30	1.22 ± 0.57	1.55 ± 0.31	3.77 ± 1.08	5.56 ± 1.22
*P* value			0.77	0.91	0.95	0.95
rs1160985						
CC	46 ± 11	17/16	1.24 ± 0.62	1.55 ± 0.34	3.73 ± 0.96	5.53 ± 1.11
CT	47 ± 8	24/40	1.27 ± 0.61	1.57 ± 0.33	3.87 ± 0.96	5.69 ± 1.02
TT	46 ± 11	13/10	1.35 ± 0.51	1.38 ± 0.40	3.57 ± 1.08	5.23 ± 1.30
*P* value			0.98	0.58	0.51	0.49
rs1064725						
TT	47 ± 9	48/60	1.26 ± 0.59	1.55 ± 0.34	3.79 ± 1.00	5.59 ± 1.11
G allele	45 ± 10	6/6	1.40 ± 0.65	1.42 ± 0.46	3.60 ± 0.85	5.29 ± 1.00
*P* value			0.39	0.32	0.79	0.65

*P* values for association between genotypes and lipids levels were obtained by using general linear model adjusted for age, sex, body mass index, and ethnicity. Values are mean ± SD

The dominant model was applied for all SNPs (rare homozygotes were grouped with heterozygotes and compared with common homozygotes), except SNPs rs405509 and rs1160985 where additive model was applied (common homozygotes vs. heterozygotes vs. rare homozygotes)

*TAG* triacylglycerol, *HDL-C* high-density lipoprotein cholesterol, *LDL-C* low-density lipoprotein cholesterol, *LPL* lipoprotein lipase, *APOE* apolipoprotein E

**Table 4:** Changes in lipid levels after dietary intervention over 16 weeks relative to baseline according to the *APOE* rs1064725 genotype

	SFA			MUFA			n-6 PUFA			P_interaction_
	TT (N = 38)	G allele (N = 3)	P _association_	TT (N = 33)	G allele (N = 3)	P _association_	TT (N = 37)	G allele (N = 6)	P _association_	
TAG	0.001 ± 1.76	0.22 ± 0.51	0.81	−0.01 ± 0.35	0.30 ± 0.32	0.15	−0.66 ± 2.05	0.04 ± 0.23	0.12	0.67
HDL-C	0.06 ± 1.70	−0.003 ± 0.14	0.87	0.003 ± 0.17	−0.06 ± 0.11	0.63	0.21 ± 2.08	0.20 ± 0.23	0.85	0.99
LDL-C	0.05 ± 1.48	2.90 ± 4.41	0.02	−0.55 ± 2.47	1.49 ± 2.70	0.27	−0.22 ± 2.24	1.99 ± 3.71	0.03	0.88

*P* values for association between genotypes and changes of means over 16 weeks with one of three diets were obtained by using general linear model adjusted for age, sex, body mass index, and ethnicity. *P* values for interaction between genotypes and changes of means over 16 weeks of intervention with one of three diets were obtained by using general linear model adjusted for age, sex, body mass index, and ethnicity. Values are mean ± SD

*TAG* triacylglycerol, *HDL-C* high-density lipoprotein cholesterol, *LDL-C* low-density lipoprotein cholesterol, *SFA* saturated fatty acids, *MUFA* monounsaturated fatty acids, *PUFA* polyunsaturated fatty acids

**Table 5:** Changes in lipid levels after dietary intervention over 16 weeks according to *LPL* rs320 genotypes

	SFA			MUFA			n-6 PUFA			P_interaction_
	TT (*N* = 21)	G allele (*N* = 20)	P_association_	TT (*N* = 11)	G allele (N = 24)	P_association_	TT (N = 24)	G allele (N = 19)	P_association_	
Total cholesterol	0.37 ± 0.60	0.29 ± 0.45	0.59	0.21 ± 2.66	−0.67 ± 2.07	0.21	−0.32 ± 1.67	−0.12 ± 0.64	0.92	0.34
TAG	−0.04 ± 2.35	0.07 ± 0.59	0.84	0.04 ± 0.50	0.01 ± 0.29	0.58	−0.07 ± 0.32	−1.19 ± 2.78	0.02	0.15
HDL-C	0.09 ± 0.15	0.01 ± 2.33	0.94	−0.07 ± 0.17	0.03 ± 0.15	0.04	0.02 ± 0.25	0.43 ± 2.91	0.78	0.75
LDL-C	0.29 ± 2.71	0.27 ± 0.40	0.95	−1.70 ± 2.85	0.16 ± 2.22	0.11	0.91 ± 2.23	−1.00 ± 2.50	0.007	0.005

*P* values for association between genotypes and changes of means over 16 weeks with one of three diets were obtained by using general linear model adjusted for age, sex, body mass index, and ethnicity. *P* values for interaction between genotypes and changes of means over 16 weeks of intervention with one of three diets were obtained by using general linear model adjusted for age, sex, body mass index, and ethnicity. Values are mean ± SD

*TAG* triacylglycerol, *HDL-C* high-density lipoprotein cholesterol, *LDL-C* low-density lipoprotein cholesterol, *SFA* saturated fatty acids, *MUFA* monounsaturated fatty acids, *PUFA* polyunsaturated fatty acids

**Fig. 1:**
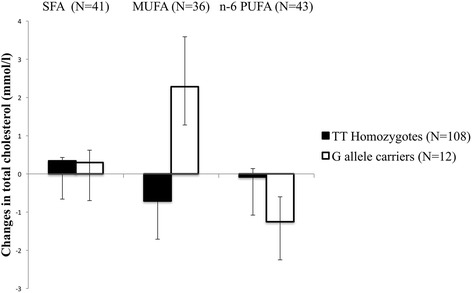
Mean (±SE) of changes in total cholesterol concentrations following three intervention diets [rich in either saturated fatty acids (SFA), monounsaturated fatty acids (MUFA), and n-6 polyunsaturated fatty acids (PUFA)] according to the *APOE* SNP rs1064725 genotype (P_interaction_ = 0.001). A general linear model analysis was performed with adjustments for age, sex, body mass index, and ethnicity. Individuals carrying the ‘TT’ genotype had lower total cholesterol levels after consuming the MUFA diet compared to the SFA or n-6 PUFA diets (P_association_ = 0.004)

At 16 weeks, after adjustment for age, sex, ethnicity and baseline BMI, a significant interaction between the *APOE* SNP rs1064725 and dietary intervention (SFA vs. MUFA vs. n-6 PUFA) on changes in fasting total cholesterol (P_interaction_ = 0.001) was observed (Fig. 1). The ‘TT’ homozygotes (*n* = 108) of SNP rs1064725 had significantly lower total cholesterol concentrations after the MUFA (*n* = 33; −0.71 ± 1.88 mmol/l) compared with the SFA (*n* = 38; 0.34 ± 0.55 mmol/l; *P* = 0.003) and n-6 PUFA-rich diets (*n* = 37; −0.08 ± 0.73 mmol/l; *P* = 0.15) (P_association_ = 0.004) (Fig. 1).

In addition, we also observed an interaction between *LPL* SNP rs320 and the dietary fat intervention (SFA vs. MUFA vs n-6 PUFA) on changes in LDL-C concentrations after 16 weeks (P_interaction_ = 0.005) (Table 5). In the n-6 PUFA diet group, the G allele carriers (*n* = 19) of the *LPL* SNP showed a reduction in LDL-C levels (−1.0 ± 2.51 mmol/l) compared to the TT genotype (*n* = 24; 0.91 ± 2.23 mmol/l) (P_association_ = 0.007). However, this interaction was not statistically significant after correction for multiple testing. None of the other SNPs showed a significant interaction on changes in lipid concentrations after the 16-week dietary intervention (Additional file 1: Tables S1-S3).

## Discussion

To our knowledge, this is the first study to investigate the effects of SNPs in both *LPL* and *APOE* genes on fasting serum lipid response after substituting SFA with MUFA or n-6 PUFA. Our findings from this retrospective analysis of the DIVAS study showed that ‘TT’ homozygotes (90% of study population) at *APOE* SNP rs1064725 had significantly lower total cholesterol concentrations after the 16-week replacement of SFA with MUFA in adults at moderate risk of CVD. Our findings indicate a greater sensitivity of this genotype group to dietary fat composition, particularly with respect to replacement of SFA with MUFA, which may have important public health implications.

Findings from cross-sectional studies are not adequate to prove the beneficial impact of a dietary component on disease prevention; therefore, data from chronic dietary intervention studies are preferable to detect changes in disease biomarkers over a period of time [37]. A dietary intervention study has shown a reduction of 51% in fasting total cholesterol in non-diabetic adults with mild abdominal obesity after two weeks of following a MUFA-rich diet (20% TE) compared to a SFA-rich diet (19% TE) [38]. In support of the beneficial effect of MUFA-rich olive oil, a Mediterranean diet supplemented with extra-virgin olive oil for 4.8 years in older adults has also been shown to reduce the incidence of major CVD events [39], which suggests the potential role of MUFA and/or nutraceuticals such as polyphenols found in extra-virgin olive oil in the prevention of CVD-related outcomes [40]. Our retrospective data analysis has demonstrated a significant interaction between *APOE* SNP rs1064725 and a MUFA-rich diet on total cholesterol levels in adults at moderate CVD risk, where the MUFA-rich diet reduced fasting total cholesterol in ‘TT’ homozygotes compared to the SFA- and n-6 PUFA-rich diets. Our finding is in line with a previous study that also showed *APOE* genotypes to modulate changes in plasma total cholesterol and LDL-C in healthy individuals after consuming MUFA- (22% TE, virgin olive oil), SFA- (20% TE), and carbohydrate- (55% TE) rich diets for 4 weeks, where levels were higher in the *E4/E3* carriers, intermediate in *E3/E3* carriers, and lower in *E3/E2* carriers [24]. Another study showed that a MUFA-rich dietary intervention (mainly olive oil) for 12 months increased the secretion of TAG-rich lipoproteins (TRL) containing apoE and decreased the secretion of those without apoE. As a result, a MUFA-rich diet shortened the residence time of very low density lipoprotein (VLDL) particles in the circulation and increased the direct clearance of TRL from the circulation (due to the enrichment of TRLs with apoE, a ligand for receptor mediated uptake), decreasing their conversion to LDLs [41]. Hence, it can be hypothesised that a MUFA-rich diet is likely to regulate the clearance rate of TRL among ‘TT’ genotype carriers of the *APOE* SNP rs1064725 via effects on TRL particle apolipoprotein composition. However, the underlying mechanism of how the ‘TT’ genotype acts differently from the ‘G’ allele on TRL metabolism in response to a MUFA-rich diet remains unclear and requires further investigation.

In our study, the common *LPL* SNP rs320 was found to modify the association between the n-6 PUFA-rich diet with changes in LDL-C levels, where the ‘G’ allele carriers had a tendency for a greater reduction in LDL-C concentrations compared to TT homozygotes. As far as the authors are aware, there are currently no studies to compare our findings with, except for one which showed that minor allele (‘G’) carriers of *LPL* SNP rs328 had lower fasting TAG concentrations when the participants had n-6 PUFA intake below 35.48% of total fat (below 35.48% of total fat median intake of LIPGENE study population) [19]. Besides *LPL*, evidence also suggests that the genetic effect of SNPs in *APOA5* and *TNFA* on lipid metabolism is modulated by n-6 PUFA [42, 43]. In mice, n-6 PUFA intake has been shown to play a role in the upregulation of genes encoding proteins involved in adipogenesis [44]. Thus, dietary n-6 PUFA may upregulate *LPL* gene expression and/or activity, leading to lower circulating lipid concentrations [19, 45]. In addition to the role of LPL in hydrolysing TRL, LPL plays a role in binding TRL (i.e. VLDL) to hepatic LDL receptors, which help to mediate the clearance of these particles [46]. This leads to a reduced conversion of VLDL to LDL, resulting in lower plasma LDL-C levels [47]. Even though the interaction between the *LPL* SNP rs320 and n-6 PUFA-rich diet on LDL-C concentrations in the current data analysis was not statistically significant after Bonferroni correction, which could be due to the small sample size, further large studies are required to explore this gene-diet interaction.

Statistically significant interactions were demonstrated in this study, however there are some limitations. The sample size was relatively small for some of the genotype groups as the genotyping was performed retrospectively, and investigation of the lipid response according to *APOE* and *LPL* SNPs was not the main objective of the DIVAS study. Compared with cross-sectional studies, randomized clinical trials are conducted with smaller sample sizes. In our study, only 120 participants out of 195 consented to genetic analysis and hence this resulted in a small sample size for the analysis. However, we were able to identify significant gene-diet interactions on total cholesterol even after Bonferroni correction. Thus, this hypothesis testing analysis has identified the need for suitably-powered dietary intervention trials using prospective genotyping to investigate the impact of dietary fat composition on plasma lipid responses according to *APOE* genotypes. A selection bias may also have existed because the participants were multi-ethnic (Asian 7% and Black 7%). However, to reduce this potential confounding effect, the analyses were adjusted for ethnicity. Furthermore, the interaction between SNP rs1064725 at *APOE* and the intervention diets on total cholesterol was still significant (*P* = 0.003) even after excluding other ethnic groups from the analysis (data not shown). One of the main strengths of our study was that it examined the effects of three types of dietary fat (isoenergetically) consumed for a long duration (16 weeks) on lipid phenotypes in a robust randomised controlled intervention study, which addressed current dietary fat recommendations. Furthermore, we used a tagSNP approach whereby all the genetic variations in the *APOE* gene have been investigated in this study.

## Conclusion

In conclusion, our study shows an interaction between *APOE* SNP rs1064725 and dietary fat intake on fasting total cholesterol concentrations, suggesting a greater sensitivity of the ‘TT’ homozygotes (90%) to dietary fat composition, with a total cholesterol lowering effect observed following substitution of SFA with MUFA but not with n-6 PUFA. However, given that the present study was conducted in a relatively small group of individuals, further large studies using prospective genotyping are required to confirm our findings.

## Additional file

Additional file 1: Table S1. Changes in lipid levels after intervention with one of three diets over 16 weeks according to *LPL* rs328 genotypes. **Table S2.** Changes in lipid levels after intervention with one of three diets over 16 weeks according to *APOE* rs405509 and rs1160985 genotypes. **Table S3.** Changes in lipid levels after intervention with one of three diets over 16 weeks according to *APOE* rs769450, rs439401, rs445925 and rs405697 genotypes. (DOCX 26 kb)

## Abbreviations

*APOE*: Apolipoprotein E
BMI: Body mass index
CVD: Cardiovascular disease
DIVAS: Dietary Intervention and VAScular function study
GLM: General linear model
HDL-C: High-density lipoprotein cholesterol
LDL-C: Low-density lipoprotein cholesterol
*LPL*: Lipoprotein lipase
MUFA: Monounsaturated fatty acids
PUFA: n-6 Polyunsaturated fatty acids
SD: Standard deviation
SFA: Saturated fatty acids
SNPs: Single-nucleotide polymorphisms
TAG: triacylglycerol
TagSNPs: Tagging SNPs
TE: Total energy
TRL: TAG-rich lipoproteins
VLDL: Very low density lipoprotein
