# Supplementary material for: Apolipoprotein E gene polymorphism modifies fasting total cholesterol concentrations in response to replacement of dietary saturated with monounsaturated fatty acids in adults at moderate cardiovascular disease risk
Source: Lipids Health Dis. 2017 Nov 23;16:222. doi: 10.1186/s12944-017-0606-3 (PMC5701425; doi:10.1186/s12944-017-0606-3)
Supplement: Additional file 1: Table S1. — Changes in lipid levels after intervention with one of three diets over 16 weeks according to LPL rs328 genotypes. Table S2. Changes in lipid levels after intervention with one of three diets over 16 weeks according to APOE rs405509 and rs1160985 genotypes. Table S3. Changes in lipid levels after intervention with one of three diets over 16 weeks according to APOE rs769450, rs439401, rs445925 and rs405697 genotypes. (DOCX 26 kb) [file 12944_2017_606_MOESM1_ESM.docx]

**Table S1:** **Changes in lipid levels after dietary intervention over 16 weeks according to *LPL* rs328 genotype**

|  | **SFA** | |  | **MUFA** | |  | **n-6 PUFA** | |  | **P_interaction_** |
| --- | --- | --- | --- | --- | --- | --- | --- | --- | --- | --- |
|  | **CC (N=31)** | **G allele (N=10)** | **P _association_** | **CC (N=24)** | **G allele (N=12)** | **P _association_** | **CC (N=34)** | **G allele (N=9)** | **P _association_** |  |
| **Total cholesterol** | 0.35± 0.59 | 0.26± 0.25 | 0.63 | -0.25± 2.26 | -0.81± 2.28 | 0.46 | -0.29± 1.42 | -0.01± 0.65 | 0.86 | 0.55 |
| **TAG** | 0.06± 1.92 | -0.12± 0.49 | 0.76 | 0.06± 0.35 | -0.09± 0.35 | 0.11 | -0.56± 1.72 | -0.62± 2.77 | 0.79 | 0.97 |
| **HDL-C** | -0.19± 1.31 | 0.82± 2.32 | 0.11 | -0.04± 0.16 | 0.07± 0.15 | 0.16 | 0.01± 1.79 | 0.97± 2.44 | 0.53 | 0.45 |
| **LDL-C** | 0.29± 2.19 | 0.23± 0.23 | 0.59 | -0.90± 2.09 | 0.82± 3.06 | 0.24 | -0.10± 2.82 | -0.15± 0.38 | 0.78 | 0.25 |

P values for association between genotypes and changes of means over 16 weeks with one of three diets were obtained by using general linear model adjusted for age, sex, body mass index, and ethnicity. P values for interaction between genotypes and changes of means over 16 weeks of intervention period with one of three diets were obtained by using general linear model adjusted for age, sex, body mass index, and ethnicity. Values are mean ± SD.

TAG; triacylglycerol, HDL-C; high-density lipoprotein cholesterol, LDL-C; low-density lipoprotein cholesterol, SFA; saturated fatty acids, MUFA; monounsaturated fatty acids, PUFA; polyunsaturated fatty acids.

**Table S2: Changes in lipid levels after dietary intervention over 16 weeks according to *APOE* rs405509 and rs1160985 genotypes**

|  | **SFA** | | |  | **MUFA** | | |  | **n-6 PUFA** | | |  | **P_interaction_** |
| --- | --- | --- | --- | --- | --- | --- | --- | --- | --- | --- | --- | --- | --- |
| ***APOE* SNP rs405509** | **GG (N=12)** | **GT (N=20)** | **TT (N=9)** | **P _association_** | **GG (N=7)** | **GT (N=26)** | **TT (N=3)** | **P _association_** | **GG (N=12)** | **GT (N=18)** | **TT (N=13)** | **P _association_** |  |
| **Total cholesterol** | 0.22± 0.65 | 0.23± 0.45 | 0.67± 0.41 | 0.12 | -0.23± 0.57 | -0.51± 2.59 | -0.12± 0.17 | 0.77 | -0.001± 0.80 | 0.05± 0.76 | -0.84± 2.00 | 0.24 | 0.55 |
| **TAG** | -0.03± 0.64 | 0.02± 2.43 | 0.07± 0.44 | 0.94 | 0.19± 0.51 | -0.01± 0.31 | -0.03± 0.49 | 0.45 | -0.52± 2.32 | -0.50± 1.71 | -0.71± 2.01 | 0.99 | 0.98 |
| **HDL-C** | -0.60± 2.12 | 0.44± 1.64 | 0.07± 0.14 | 0.32 | -0.002± 0.11 | -0.01± 0.18 | 0.02± 0.03 | 0.98 | 0.63± 2.12 | -0.31± 1.75 | 0.55± 2.03 | 0.48 | 0.18 |
| **LDL-C** | 0.20± 0.55 | 0.61± 1.88 | -0.29± 2.91 | 0.60 | -1.06± 1.73 | 0.22± 2.03 | -3.88± 4.56 | 0.01 | -1.11± 3.47 | 0.56± 2.12 | 0.40± 1.77 | 0.23 | 0.10 |
|  | | | | | | | | | | | | | |
| ***APOE SNP* rs1160985** | **CC (N=11)** | **CT (N=19)** | **TT (N=11)** | **P _association_** | **CC (N=8)** | **CT (N=22)** | **TT (N=6)** | **P _association_** | **CC (N=14)** | **CT (N=23)** | **TT (N=6)** | **P _association_** | **P_interaction_** |
| **Total cholesterol** | 0.64± 0.38 | 0.34± 0.54 | -0.02± 0.46 | 0.01 | 0.05± 0.32 | -0.66± 2.82 | -0.20± 0.66 | 0.78 | -0.67± 1.94 | 0.01± 0.81 | -0.17± 0.90 | 0.36 | 0.40 |
| **TAG** | 0.04± 0.41 | -0.001± 2.50 | 0.03± 0.66 | 0.95 | 0.05± 0.29 | -0.05± 0.33 | 0.26± 0.55 | 0.27 | -0.66± 1.93 | -0.30± 1.64 | -1.51± 3.07 | 0.52 | 0.62 |
| **HDL-C** | 0.05± 0.14 | 0.49± 1.68 | -0.70± 2.21 | 0.23 | -0.01± 0.18 | 0.002± 0.17 | -0.01± 0.13 | 0.94 | 0.54± 1.94 | 0.06± 2.18 | -0.03± 0.21 | 0.84 | 0.57 |
| **LDL-C** | -0.14± 2.61 | 0.72± 1.92 | -0.02± 0.43 | 0.65 | -1.36± 3.21 | 0.21± 2.23 | -1.24± 1.95 | 0.38 | 0.69± 1.81 | -0.01± 2.79 | -1.32± 2.72 | 0.25 | 0.44 |

P values for association between genotypes and changes of means over 16 weeks with one of three diets were obtained by using general linear model adjusted for age, sex, body mass index, and ethnicity. P values for interaction between genotypes and changes of means over 16 weeks of intervention period with one of three diets were obtained by using general linear model adjusted for age, sex, body mass index, and ethnicity. Values are mean ± SD.

TAG; triacylglycerol, HDL-C; high-density lipoprotein cholesterol, LDL-C; low-density lipoprotein cholesterol, SFA; saturated fatty acids, MUFA; monounsaturated fatty acids, PUFA; polyunsaturated fatty acids.

**Table S3: Changes in lipid levels after dietary intervention over 16 weeks according to *APOE* rs769450, rs439401, rs445925 and rs405697 genotypes**

|  | **SFA** | |  | **MUFA** | |  | **n-6 PUFA** | |  | **P_interaction_** |
| --- | --- | --- | --- | --- | --- | --- | --- | --- | --- | --- |
| ***APOE* SNP rs769450** | **GG (N=12)** | **A allele (N=29)** | **P _association_** | **GG (N=11)** | **A allele (N=25)** | **P _association_** | **GG (N=21)** | **A allele (N=22)** | **P _association_** |  |
| **Total cholesterol** | 0.56± 0.46 | 0.23± 0.53 | 0.06 | -0.22± 0.74 | -0.53± 2.74 | 0.74 | -0.55± 1.66 | 0.06± 0.81 | 0.14 | 0.34 |
| **TAG** | 0.05± 0.39 | 0.001± 2.04 | 0.74 | 0.01± 0.37 | 0.02± 0.35 | 0.83 | -0.48± 1.60 | -0.65± 2.22 | 0.86 | 0.92 |
| **HDL-C** | 0.05± 0.13 | 0.05± 1.98 | 0.94 | -0.06± 0.19 | 0.03± 0.14 | 0.26 | 0.72± 2.21 | -0.26± 1.57 | 0.15 | 0.21 |
| **LDL-C** | -0.17± 2.49 | 0.48±1.59 | 0.51 | -0.77± 3.24 | -0.15± 2.10 | 0.91 | 0.36± 3.34 | -0.22± 1.47 | 0.39 | 0.51 |
|  | | | | | | | | | | |
| ***APOE* SNP rs439401** | **CC (N=19)** | **T allele (N=22)** | **P _association_** | **CC (N=17)** | **T allele (N=19)** | **P _association_** | **CC (N=14)** | **T allele (N=27)** | **P _association_** | **P_interaction_** |
| **Total cholesterol** | 0.13± 0.48 | 0.50± 0.51 | 0.03 | -0.23± 0.59 | -0.60± 3.07 | 0.77 | -0.06± 0.64 | -0.26± 1.55 | 0.77 | 0.63 |
| **TAG** | -0.42± 1.79 | 0.38± 1.55 | 0.19 | -0.03± 0.39 | 0.06± 0.32 | 0.23 | -0.001± 0.94 | -0.87± 2.27 | 0.38 | 0.13 |
| **HDL-C** | 0.02± 2.47 | 0.08± 0.17 | 0.94 | 0.02± 0.18 | -0.02± 0.15 | 0.91 | 0.001± 2.99 | 0.33± 1.38 | 0.66 | 0.92 |
| **LDL-C** | -0.36± 2.01 | 0.79± 1.71 | 0.09 | -0.37± 0.68 | -0.35± 3.44 | 0.85 | -.043± 2.98 | 0.33± 2.40 | 0.34 | 0.59 |
|  | | | | | | | | | | |
| ***APOE* SNP rs445925** | **GG (N=31)** | **A allele (N=8)** | **P _association_** | **GG (N=26)** | **A allele (N=9)** | **P _association_** | **GG (N=34)** | **A allele (N=9)** | **P _association_** | **P_interaction_** |
| **Total cholesterol** | 0.35± 0.48 | 0.26± 0.76 | 0.89 | -0.39± 2.68 | -0.43± 0.79 | 0.99 | -0.22± 1.42 | -0.26± 0.70 | 0.74 | 0.98 |
| **TAG** | 0.08± 1.96 | -0.22± 0.49 | 0.75 | 0.07± 0.34 | -0.05± 0.34 | 0.47 | -0.69± 2.15 | -0.09± 0.16 | 0.65 | 0.68 |
| **HDL-C** | 0.31± 1.32 | -0.84± 2.49 | 0.12 | 0.02± 0.14 | -0.05± 0.22 | 0.65 | 0.26± 2.17 | -0.02± 0.22 | 0.89 | 0.33 |
| **LDL-C** | 0.27± 2.21 | 0.28± 0.64 | 0.75 | -0.53± 2.82 | 0.14± 1.79 | 0.66 | -0.24± 2.29 | 1.21± 3.19 | 0.18 | 0.61 |
|  | | | | | | | | | | |
| ***APOE* SNP rs405697** | **GG (N=25)** | **A allele (N=16)** | **P _association_** | **GG (N=24)** | **A allele (N=12)** | **P _association_** | **GG (N=21)** | **A allele (N=21)** | **P _association_** | **P_interaction_** |
| **Total cholesterol** | 0.26± 0.58 | 0.45± 0.44 | 0.39 | -0.25± 2.24 | -0.81± 2.33 | 0.60 | -0.04± 0.73 | -0.39± 1.67 | 0.28 | 0.61 |
| **TAG** | -0.36± 1.53 | 0.60± 1.80 | 0.11 | 0.02± 0.39 | -0.002± 0.27 | 0.91 | -0.35± 1.82 | -0.46± 1.53 | 0.87 | 0.20 |
| **HDL-C** | 0.05± 2.11 | 0.06± 0.17 | 0.97 | 0.03± 0.15 | -0.06± 0.17 | 0.15 | 0.05± 2.41 | 0.36± 1.54 | 0.73 | 0.87 |
| **LDL-C** | 0.22± 2.46 | 0.36± 0.36 | 0.98 | -0.34± 1.48 | -0.40± 4.09 | 0.94 | -0.21± 3.49 | 0.29± 1.37 | 0.52 | 0.86 |

P values for association between genotypes and changes of means over 16 weeks with one of three diets were obtained by using general linear model adjusted for age, sex, body mass index, and ethnicity*.* P values for interaction between genotypes and changes of means over 16 weeks of intervention period with one of three diets were obtained by using general linear model adjusted for age, sex, body mass index, and ethnicity. Values are mean ± SD. TAG; triacylglycerol, HDL-C; high-density lipoprotein cholesterol, LDL-C; low-density lipoprotein cholesterol, SFA; saturated fatty acids, MUFA; monounsaturated fatty acids, PUFA; polyunsaturated fatty acid.
